# Proteomic analysis of differentially expressed proteins involved in ethylene-induced chilling tolerance in harvested banana fruit

**DOI:** 10.3389/fpls.2015.00845

**Published:** 2015-10-15

**Authors:** Taotao Li, Ze Yun, Dandan Zhang, Chengwei Yang, Hong Zhu, Yueming Jiang, Xuewu Duan

**Affiliations:** ^1^Key Laboratory of Plant Resources Conservation and Sustainable Utilization/Guangdong Provincial Key Laboratory of Applied Botany, South China Botanical Garden, Chinese Academy of SciencesGuangzhou, China; ^2^College of Life Science, University of Chinese Academy of SciencesBeijing, China; ^3^Guangdong Provincial Key Laboratory of Biotechnology for Plant Development, College of Life Sciences, South China Normal UniversityGuangzhou, China

**Keywords:** banana, ethylene, chilling tolerance, energy metabolism, methionine salvage cycle

## Abstract

To better understand the mechanism involved in ethylene-induced chilling tolerance in harvested banana fruit, a gel-based proteomic study followed by MALDI-TOF-TOF MS was carried out. Banana fruit were treated with 500 ppm ethylene for 12 h and then stored at 6°C. During cold storage, the chilling tolerance was assessed and the proteins from the peel were extracted for proteomic analysis. It was observed that ethylene pretreatment significantly induced the chilling tolerance in harvested banana fruit, manifesting as increases in maximal chlorophyll fluorescence (*Fv/Fm*) and decreased electrolyte leakage. Sixty-four proteins spots with significant differences in abundance were identified, most of which were induced by ethylene pretreatment during cold storage. The up-regulated proteins induced by ethylene pretreatment were mainly related to energy metabolism, stress response and defense, methionine salvage cycle and protein metabolism. These proteins were involved in ATP synthesis, ROS scavenging, protective compounds synthesis, protein refolding and degradation, and polyamine biosynthesis. It is suggested that these up-regulated proteins might play a role in the ethylene-induced chilling tolerance in harvested banana fruit.

## Introduction

Banana is a typical climacteric fruit. The harvested fruit undergo a rapid ripening process, resulting in a short shelf life. Cold storage is effective in extending the postharvest life of banana fruit. However, the fruit are susceptible to chilling injury (CI), manifesting as peel browning, pitting, and a failure to soften when the storage temperature is lower than 12°C (Jiang et al., 2004). To alleviate chilling injury, different strategies such as heat treatment, NO or gamma-aminobutyric acid (GABA) pretreatment, have been applied in harvested banana before cold storage (Wang et al., 2013, 2014; Ma et al., 2014).

Ethylene plays a key role in fruit ripening, acting as a signal in fruit ripening-associated process. Two systems (system-1 and system-2) are involved in ethylene biosynthesis. System-1 is responsible for producing the basal level of ethylene synthesized by all plant tissues, including non-climacteric fruit, while system-2 functions during ripening of climacteric fruit, such as banana and tomato, as well as petal senescence (reviewed by Lelievre et al., 2007). Ethylene synthesis is essential for climacteric fruit ripening (reviewed by Lin et al., 2009). The transition from system-1 to system-2 results in a sharp increase in climacteric fruit ethylene, which is considered to be the initiator of changes in color, texture, aroma and flavor, and other biochemical and physiological attributes (reviewed by Alexander and Grierson, 2002).

It should be noted that ethylene is also involved in a cold stress response in some fruits. Studies have shown that exogenous ethylene treatment accelerates CI symptoms in avocado (Pesis et al., 2002) and plum (Candan et al., 2011), but alleviated the CI-related disorders in nectarine (Dong et al., 2001). Application of 1-MCP (1-methylcyclopropene), an inhibitor of ethylene perception, further confirmed the role of ethylene in the response of fruit to low temperature. It has been found that 1-MCP treatment inhibited the development of chilling injury in apple (Fan and Mattheis, 1999), avocado (Pesis et al., 2002), pineapple (Selvarajah et al., 2001), plum (Candan et al., 2011), and loquat (Cao et al., 2010). Nevertheless, chilling injury symptoms of banana (Jiang et al., 2004), “Shamouti” orange (Porat et al., 1999), and peach (Fan et al., 2002) were accelerated by 1-MCP treatment. These results suggest that the response of different species of fruits to ethylene under low temperature stress varies and that the mechanisms of ethylene action on chilling injury and development in various harvested fruits is relatively complex. Our preliminary study has shown that ethylene pretreatment was efficient in alleviating chilling injury symptoms in harvested banana fruit. However, the underlying mechanism involved in the induced-chilling tolerance by ethylene treatment in the fruit remains largely unknown.

Proteomics provides an important approach to elucidate the molecular mechanisms involved in development and stress response in plants at the protein level. Recently, proteomics approaches have been used to identify a large number of responsive proteins to low temperature stress in harvested fruit. Vega-Garcia et al. (2010) identified two proteins (thioredoxin peroxidase and glycine-rich RNA-binding protein) related to cold stress in tomato fruit which regulate redox status and gene expression, respectively. A proteomic study of chilling injury in tomato also revealed that some proteins related to defensive response, uncoupling of photosynthetic processes and protein degradation machinery played an important role in chilling tolerance (Sanchez-Bel et al., 2012b). Dagar et al. (2010) pointed out the potential role of thaumatin-like proteins against chilling injury in harvested peach fruit. Sánchez-Bel et al. (2012a) found that proteins linked to the ascorbate-glutathione cycle, glycolysis, Calvin cycle, and Krebs cycle were inhibited in chilled bell pepper fruit. In the case of banana, several studies have been performed on the proteome of banana meristem and fruit. Toledo et al. (2012) compared the proteins of banana pulp at the ripening pre-climacteric and climacteric stages and identified six increased proteins (at least 2-fold differences in abundance), including two chitinases, one isoflavone reductase, one S-Adenosyl-L-homocysteine hydrolase, and two heat shock proteins. Carpentier et al. (2007) investigated the acclimation mechanism of banana meristems to osmotic stress and determined some proteins involved in energy metabolism and stress adaptation were associated with the dehydration-tolerance. Furthermore, Vanhove et al. (2015) characterized the Hsp70 family during osmotic stress in banana meristems and identified a specific osmotic responsive cytoplasmic Hsp70 isoform (the Hsp70 paralog 2). In addition, Vanhove et al. (2012) analyzed the banana biodiversity for drought tolerance by proteomics analysis and found that the respiration, metabolism of ROS and several dehydrogenases involved in NAD/DADH homeostasis play an important role in drought tolerance. Unfortunately, no proteomic information on how ethylene is involved in chilling tolerance in harvested banana fruit is available.

In this study, comparative a proteomic analysis was performed to investigate the differently expressed proteins between the ethylene treated and non-ethylene-treated banana fruit during cold storage, these findings will offer new insight into ethylene effects on chilling tolerance.

## Materials and methods

### Plant material and treatment

Green mature banana (*Musa* spp., AAA group cultivar “Brazil”) fruit (approximately 110 days after anthesis) were harvested from an orchard in Huizhou city, Guangdong province, China and then transported to the laboratory within 6 h. Banana fruit fingers were cut, then dipped in 0.1% Sportak® (prochloraz, Bayer) fungicide solution for 3 min to control decay, and finally allowed to air-dry. Fruit with uniform shape, color and size were selected and randomly divided into four groups. Each group consisted of three subgroups (biological replicates), with each subgroup containing 18 fruits. Two groups were treated with 500 ppm C_2_H_4_ in sealed jars for 12 h at 24°C. The other two groups without ethylene treatment were used as controls. After the treatment, fruit were packed into 0.03 mm polyethylene bags (3 fruits per bag) and then stored at 6°C and 85% RH (relative humidity) in darkness. After 1 and 4 days of storage, physiological parameters (chilling injury index, color, chlorophyll fluorescence parameter and relative electrolyte leakage) were measured. Mixed peel tissues from the middle section of 18 fruits for each subgroup were collected, frozen in liquid nitrogen and stored at −80°C for protein extraction and analysis. The replicates and treatments in this study are shown in Figure S1.

### Physiological parameters measurements

Chilling injury was evaluated by assessing the browning or pitting extent of banana peel, using five scales: 0, no browning or pitting; 1, slightly browning or pitting; 2, less than 1/2 surface showing browning or pitting; 3, 1/2-3/4 surface showing browning or pitting; 4, severely browning or pitting. Chilling injury index was calculated as (chilling injury scale × number of each scale)/total number of fruit.

Chlorophyll fluorescence of banana peel was measured with a portable chlorophyll fluorometer (PAM 2100, Walz, Germany). PSII quantum yield (Fv/Fm) was used to express chlorophyll fluorescence parameter of the banana peel.

Membrane permeability was presented by relative electrolyte leakage, which was determined by the method of Huang et al. (2012).

### Protein extraction from banana peel

Ten grams of banana peel tissue from each subgroup were used for protein extraction according to the method of Toledo et al. (2012) with minor modifications. The samples were resuspended in 20 mL of ice-cold extraction buffer (0.5 M Tris, 0.1 M KCl, 0.7 M sucrose, 50 mM EDTA, 1% PVP, 0.2% (v/v) 2-mercaptoethanol, and 1% PMSF, pH 7.5), and then the protein was extracted using a phenol extraction protocol. After drying with a vacuum pump, the resulting pellet was resuspended in 0.3 mL isoelectric focusing (IEF) solubilization buffer (8 M urea, 4% CHAPS, 65 mM DTT, and 0.2% ampholine pH 3-10). The concentrations of the proteins were determined using the Bio-Rad Protein Assay kit (Bio-Rad, USA).

### 2-d and image analysis

Two-dimensional gel electrophoresis was carried out with 2 mg of banana protein according to Yun et al. (2010) using 17 cm IPG strip (pH 5–8, BioRad). Briefly, the IEF was performed on a Bio-Rad apparatus using 250 V for 30 min, rapid ramping at 1000 V for 1 h, then ramping to 8000 V for 5 h and maintained at 8000 V until 80000 Vh was reached. Then, the strips were equilibrated orderly in two buffers based on equilibration buffer (0.375 M Tris-HCl, pH 8.8, 6 M urea; 5% glycerol and 2% SDS) for 15 min under moderate shaking. Buffer 1 contained 0.2 mg dithiothreitol (DTT) dissolved in 10 mL equilibration buffer, whereas buffer 2 contained 250 mg iodoacetamide in 10 mL equilibration buffer. Following the second dimension SDS-PAGE (12% polyacrylamide gels) at 100 V for 0.5 h, followed by 200 V for 5 h, the gels were fixed for 1 h with 40% ethanol and 10% acetic acid in ultrapure water, washed with ultrapure water and stained using Commassie blue according to the method of Yun et al. (2010). At least three gels of independent biological replicates were run for each sample. After washing with ultrapure water, the image was scanned using MagicScan and analyzed by PDQuest 2-D analysis software (version 8.0, Bio-Rad, USA) according to the method of Yun et al. (2010). Briefly, to obtain the highest gel matching, an automated and manual matching function was used in lieu of the automated routine. The data were normalized using total quantity of valid spots based on the corresponding gel to account for quantitative variations in intensity of protein spots between samples. The normalized intensity of spots on three independent biological replicate 2D gels was averaged, and then SPSS software 13.0 (SPSS Inc., Chicago, IL, USA) was used to determine whether the relative change was statistically significant between samples. Spots with more than a 2-fold differential accumulation in comparison to those from other samples were excised and used for protein identification.

### In-gel protein digestion and protein identification by lc-ms/ms

In-gel digestion and identification were performed as described by Yun et al. (2010) with minor modifications. In brief, the differently expressed spots were manually excised from the gel and then destained three times with 500 μL of 25 mM NH_4_HCO_3_ in 50% acetonitrile for 60 min. After wash with H_2_O, the supernatant was discarded, and then the gel slices were incubated with acetonitrile for dehydration. After dehydration, the gels were treated with 10 mM DTT for 1 h before 55 mM iodoacetamide (IAM) was added for 45 min. Trypsin solution (10 ng/μL in 25 mM NH_4_HCO_3_) was used to cover the above mixture. After 30 min on ice, the excess trypsin solution was removed by the addition of 25 μL of 25 mM NH_4_HCO_3_. The mixture was then incubated at 37°C overnight. Finally, 5% formic acid was used to stop the reaction.

For LC-MS/MS, 1 μL of each sample was spotted onto the Anchor chip target plate and then dried at 25°C. After the addition of 0.1 μL HCCA (α-cyano-4-hydroxycinnamicacid) matrix solution, the target plate was placed in MALDI-TOF/TOF (Applied Biosystems, Framingham, MA, USA). MS spectra were acquired using the positive ion reflector mode with resolution ratio at 50,000 over the full-scan spectrum. The five most abundant precursor ions were selected for MS/MS scans. All acquired spectra of samples were processed using Flex Analysis 3.3 software (Bruker). The MS and MS/MS spectra were combined using BioTools 3.0 software and search with Mascot software 2.3.02 against Musaceae database with 36505 sequences (http://banana-genome.cirad.fr/home). The following parameters were used for database searches: Trypsin, Peptide Mass Tolerance 100 ppm, Fragment Mass Tolerance 0.6 Da, Miss Cleavages 1, Carbamidomethyl (C) as a fixed modification, and Oxidation (M), Gln->pyro-Glu (N-term Q), and Deamidated (NQ) as variable modification. Protein candidates provided by the combined PMF and MS/MS search were considered as valid when the global Mascot score was greater than Significance Score (58) with a significance level of *e* < 0.05. All the peptide matches for each spot were provided in Table S1.

### Statistical analysis

Experiments were conducted with three replicates. Physiological parameters were tested by analysis of variance using SPSS (Version 13.0). Least significant differences (LSDs) were calculated to compare significant effects at 5% level.

## Results

### Physiological parameters

**Figure 1:**
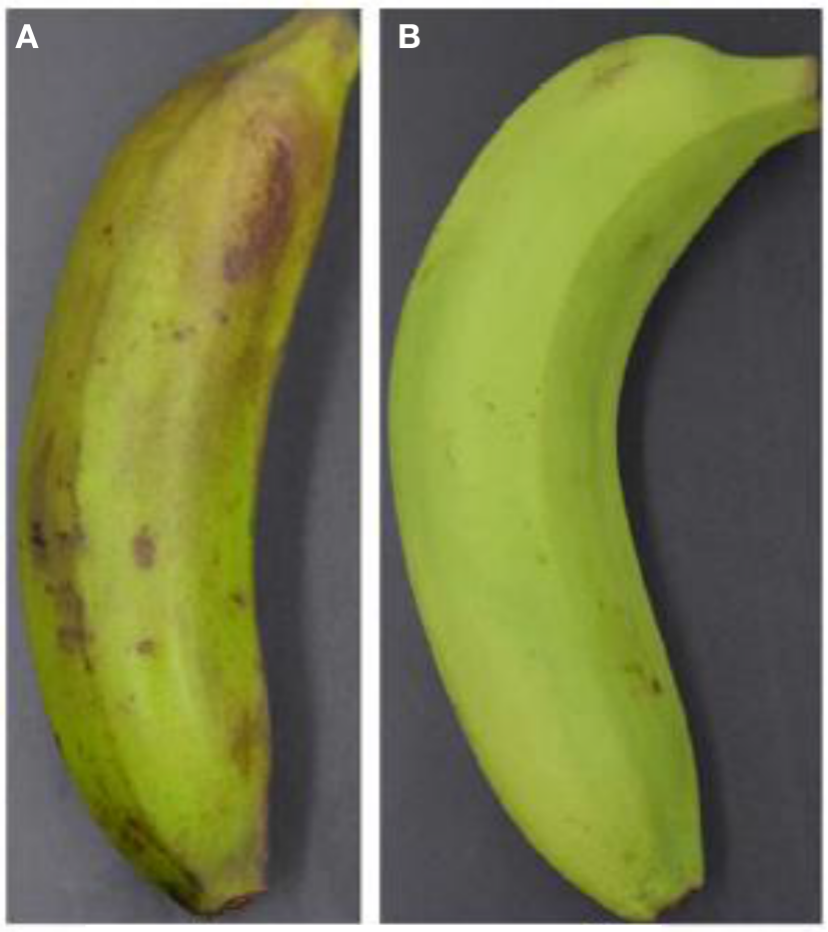
**Visual appearance of banana fruit treated with ethylene after 4 days of storage at 6°C**. **(A)**, control and **(B)**, ethylene treatment.

**Figure 2:**
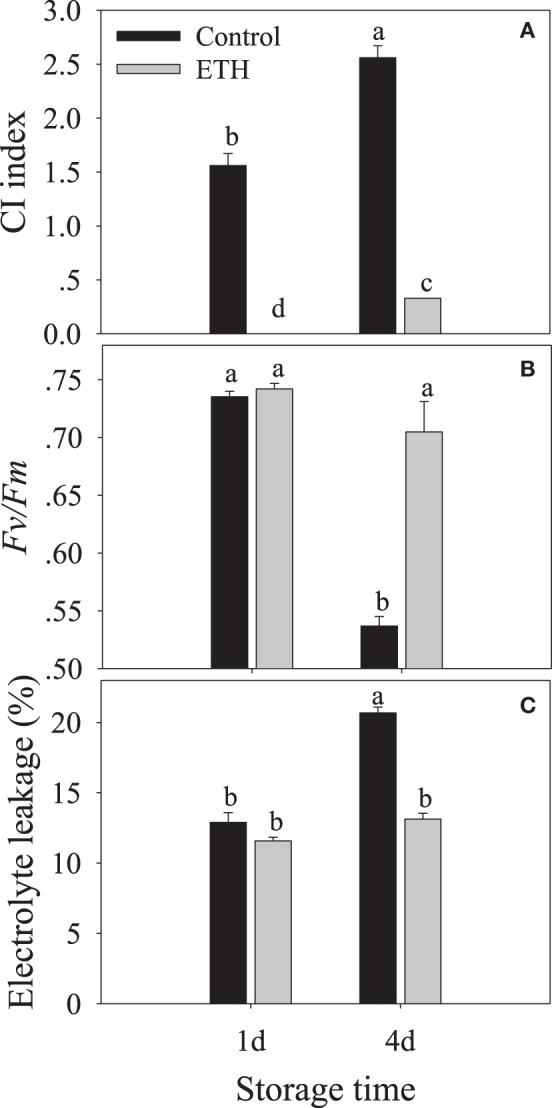
**Physiological response of the ethylene-treated banana fruit to low temperature**. Banana fruit were treated with 500 ppm ethylene for 12 h and then stored at 6°C. **(A)**, CI index; **(B)**, Fv/Fm, and **(C)**, electrolyte leakage. Data are presented as means ± standard errors (*n* = 3). Different letters represented significant difference of the values (*p* < 0.05).

After 1 days of storage at 6°C, control fruit only appeared to have slight CI symptoms while fruit treated with ethylene showed no CI development. Ethylene treatment significantly inhibited the development of chilling injury (Figure 1). After 4 days of storage, the CI indices were 2.56 and 0.33 for the control and ethylene-treated fruit, respectively (Figure 2A).

There was no significant difference in *Fv/Fm* between the control and ethylene-treated fruit after 1 days of storage at 6°C. After 4 days of storage, the *Fv/Fm* in the control fruit decreased to 0.53, while it in the ethylene-treated fruit it was 0.71 (Figure 2B).

The relative electrolyte leakage from peel tissue had an initial value of 8.6%, and then increased with the development of chilling injury. After 4 days of storage, the relative electrolyte leakage in the ethylene-treated fruit was significantly lower than that in control fruit (Figure 2C).

### Proteomic analysis of banana fruit subjected to chilling injury

**Figure 3:**
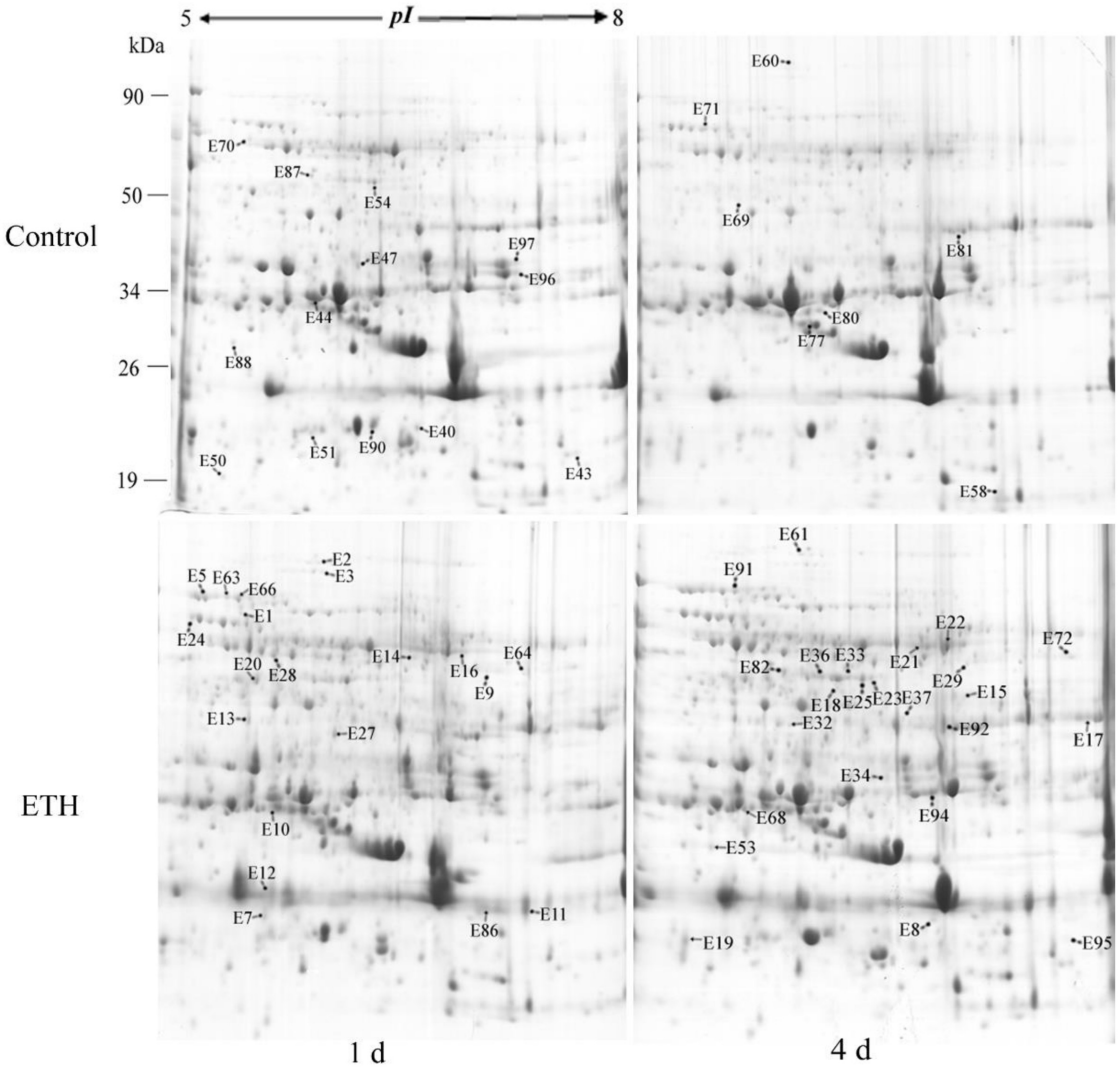
**Representative two-dimensional electrophoresis maps of banana fruit**. 2-D gels show total protein from the control and ethylene-treated banana fruit after 1 and 4 days of storage at 6°C. The locations of differentially expressed proteins successfully identified are labeled.

**Figure 4:**
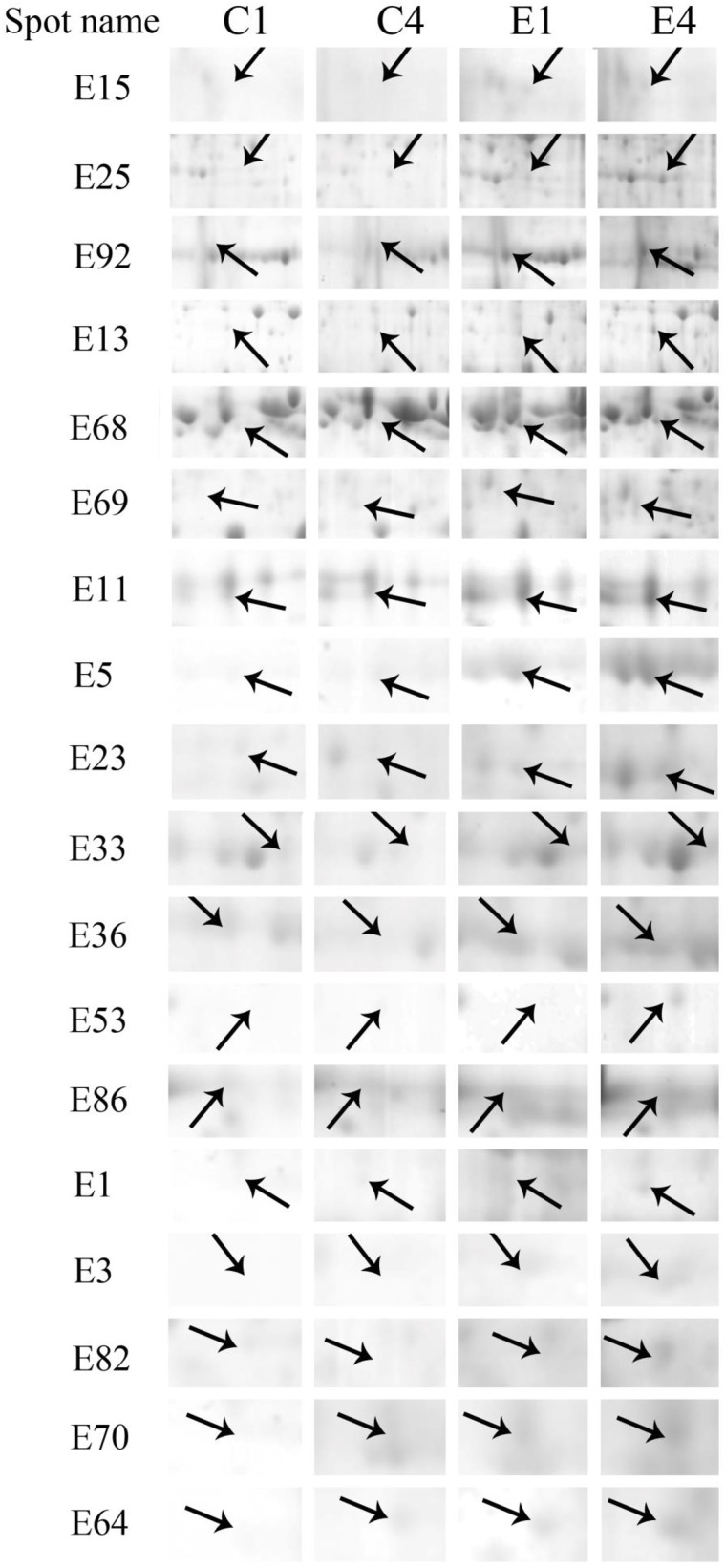
**Close-up view of some significant differentially expressed proteins marked in Figure 3**. C1, C4, E1, and E4 were the control fruit after 1 and 4 days of cold storage and the ethylene-treated fruit after 1 and 4 days of cold storage, respectively. Some typical spots with significantly differential accumulation patterns are indicated by arrow heads. Their sample names followed the manuscript annotation.

After IEF, PAGE electrophoresis and CBB staining, more than 700 reproducible spots were detected by PDQuest 2-D analysis software (Figure 3 and Figure S2). Of these 700 spots, 76 spots exhibited more than 2-fold differences in abundance when ethylene-treated fruit were compared to the control fruit after 1 or 4 days storage at 6°C. Using MALDI-TOF/TOF MS analysis, 64 spots were identified successfully (Figure 3). Some of the up-regulated proteins induced by the ethylene treatment are shown in Figure 4.

### Functional classification and expression patterns of the identified proteins

**Figure 5:**
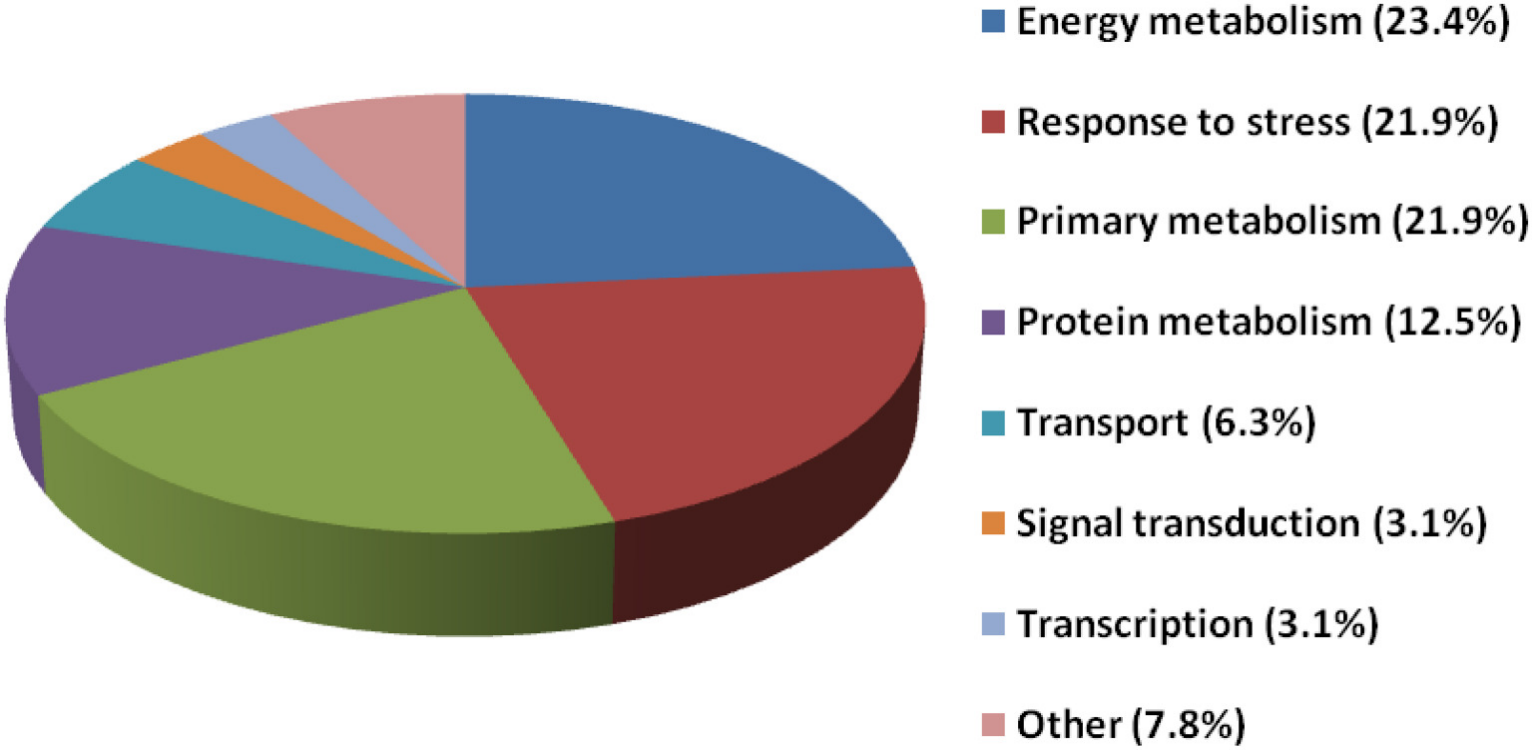
**Functional classification of differentially expressed proteins**. Differentially expressed proteins from the control and ethylene-treated banana fruit after 1 and 4 days of storage at 6°C were classified using Blast2Go.

The sixty-four proteins with differential abundances were classified into eight functional classes using Blast2Go software (Figure 5 and Table 1). The majority of these proteins came from three major classes, including energy metabolism, response to stress and defense, and primary metabolism, which accounted for 23.4, 21.9, and 21.9% of the differential proteins, respectively.

**Table 1:** **Differentially expressed proteins with functional categorization and quantitative analysis from the ethylene-treated and non-ethylene-treated (control) banana fruit after 1 and 4 days of storage at 6°C**.

**Sample name**	**Protein accumulation**	**Protein ID**	**Protein description**	**Mr (kDa)/IP**	**Pep count**	**Protein score**
**ENERGY METABOLISM**						
E15	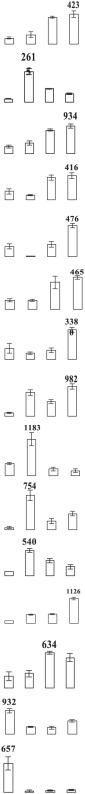	GSMUA_Achr5P25490_001	Fructose-bisphosphate aldolase cytoplasmic isozyme	36.0/6.78	17	184
E17		GSMUA_AchrUn_randomT11210_001	Malate dehydrogenase, mitochondrial	36.6/9.29	7	84.4
E27		GSMUA_Achr6P25770_001	Putative carboxyvinyl-carboxyphosphonate phosphorylmutase	33.4/5.98	10	195
E32		GSMUA_Achr6P02650_001	lactate/malate dehydrogenase	43.8/8.27	8	109
E37		GSMUA_Achr4P21920_001	Malate dehydrogenase	35.8/6.36	8	84.6
E72		GSMUA_Achr11P21550_001	Fumarate hydratase 1	53.8 /8.24	16	152
E92		GSMUA_Achr4P21920_001	Malate dehydrogenase	35.8/6.36	18	139
E13		GSMUA_Achr5P25000_001	Pyruvate dehydrogenase E1 component subunit beta	45.2/7.53	12	281
E80		GSMUA_Achr6P02850_001	Probable ATP synthase 24 kDa subunit, mitochondrial	28.1/7.56	13	221
E21		GSMUA_Achr3P06170_001	Aldehyde dehydrogenase family 2 member B7	58.2/7.31	15	102
E24		GSMUA_AchrUn_randomP08730_001	RuBisCO large subunit-binding protein subunit alpha	61.0/5.05	20	357
E25		GSMUA_Achr4P33150_001	Phosphoglycerate kinase	50.2/9.23	14	90.7
E71		GSMUA_Achr9P23240_001	RuBisCO large subunit-binding protein subunit beta	65.4/5.86	37	568
E40		GSMUA_Achr9P24590_001	Cytochrome b6-f complex iron-sulfur subunit	24.3/8.48	11	71.4
E50		GSMUA_Achr5P28730_001	Thylakoid lumenal 15 kDa protein 1	23.3/7.19	11	91.3
**STRESS RESPONSE AND DEFENSE**						
E10	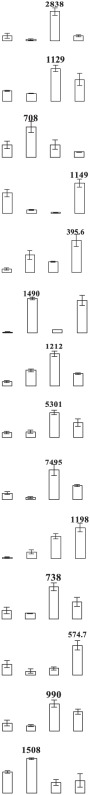	GSMUA_Achr5P07280_001	L-ascorbate peroxidase	27.5/5.2	15	382
E68		GSMUA_Achr5P07280_001	L-ascorbate peroxidase	27.5/5.2	17	61.5
E77		GSMUA_Achr11P20440_001	Glutathione S-transferase 3	22.6/5.05	5	218
E51		GSMUA_Achr2P02380_001	Superoxide dismutase [Cu-Zn]	15.3/5.78	6	129
E69		GSMUA_Achr5P20330_001	Peroxidase 5	28.3/6.5	6	91.1
E19		GSMUA_Achr10P22170_001	Peroxiredoxin-2C	13.4/4.91	4	91.9
E8		GSMUA_Achr6P31470_001	Thaumatin-like protein	20.3/4.98	4	202
E11		GSMUA_Achr6P31470_001	Thaumatin-like protein	20.3/4.98	7	327
E12		GSMUA_Achr6P31470_001	Thaumatin-like protein	20.3/4.98	5	354
E5		GSMUA_Achr8P20830_001	Heat shock cognate 70 kDa protein	71.3/4.83	20	157
E63		GSMUA_Achr8P20830_001	Heat shock cognate 70 kDa protein	71.3/4.83	34	331
E91		GSMUA_Achr8P20830_001	Heat shock cognate 70 kDa protein	71.3/4.83	19	171
E66		GSMUA_Achr8P20830_001	Heat shock cognate 70 kDa protein	71.3/4.83	35	274
E81		GSMUA_Achr11P25060_001	stress responsive protein, putative	32.4/9.4	3	145
**PRIMARY METABOLISM**						
E18	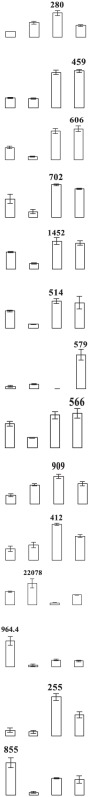	GSMUA_Achr1P12930_001	S-adenosylmethionine synthase 1	51.8/5.94	16	246
E23		GSMUA_Achr2P07970_001	S-adenosylmethionine synthase 5	43.7/5.88	10	204
E33		GSMUA_Achr5P20990_001	S-adenosylmethionine synthase	43.7/5.91	21	458
E36		GSMUA_Achr1P15960_001	S-adenosylmethionine synthase 2	43.6/5.54	5	82.8
E54		GSMUA_Achr2P07970_001	S-adenosylmethionine synthase 5	43.7/5.88	25	77.1
E87		GSMUA_Achr1P15960_001	S-adenosylmethionine synthase 2	43.6/5.54	13	227
E53		GSMUA_Achr2P08880_001	1,2-dihydroxy-3-keto-5-methylthiopentene dioxygenase 2	23.4/4.92	11	285
E88		GSMUA_Achr2P08880_001	1,2-dihydroxy-3-keto-5-methylthiopentene dioxygenase 2	23.4/4.92	13	286
E28		GSMUA_Achr9P23930_001	UTP–glucose-1-phosphate uridylyltransferase	51.5/5.54	18	377
E29		GSMUA_Achr5P00340_001	Putative Uncharacterized aminotransferase y4uB	55.6/7.38	8	121
E44		GSMUA_AchrUn_randomP12350_001	Putative Acidic endochitinase	19.6/4.86	2	172
E97		GSMUA_Achr3P32070_001	Glutamate synthase [NADH]	219.0/7.35	53	81.8
E9		GSMUA_Achr4P09600_001	Biotin carboxylase 1	58.6/8.0	14	303
E96		ITC1587_Bchr4_T11514	|patatin group a-3-like	85.6/7.67	16	70.5
**PROTEIN METABOLISM**						
E2	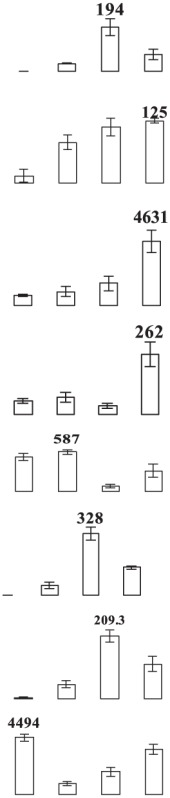	GSMUA_Achr6P16670_001	Chaperone protein ClpB 2	105.7/6.55	18	207
E61		GSMUA_Achr6P16670_001	Chaperone protein ClpB 2	105.7/6.55	15	114
E86		GSMUA_Achr2P21150_001	Peptidyl-prolyl cis-trans isomerase cyp20-2 protein	27.0/9.71	15	137
E95		GSMUA_Achr6P16580_001	Peptidyl-prolyl cis-trans isomerase cyp20-2 protein	29.6/9.75	18	183
E43		GSMUA_Achr7P22790_001	FK506-binding protein 2-1	16.2/8.22	6	89.5
E1		GSMUA_Achr7P04590_001	Cell division protease ftsH homolog 2	57.3/4.98	16	288
E3		GSMUA_Achr3P18630_001	ATP-dependent Clp protease ATP-binding subunit clpA homolog CD4B	100.1 /6.6	13	77.6
E90		GSMUA_Achr6P16740_001	Eukaryotic translation initiation factor 5A	17.6/5.87	9	214
**SIGNAL TRANSDUCTION**						
E82	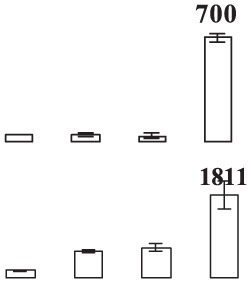	GSMUA_Achr4P15750_001	IAA-amino acid hydrolase ILR1-like 1	47.6/5.8	10	63.7
E16		GSMUA_Achr6P21830_001	Putative Septum-promoting GTP-binding protein 1	30.2/9.5	18	65.9
**TRANSPORT**						
E70	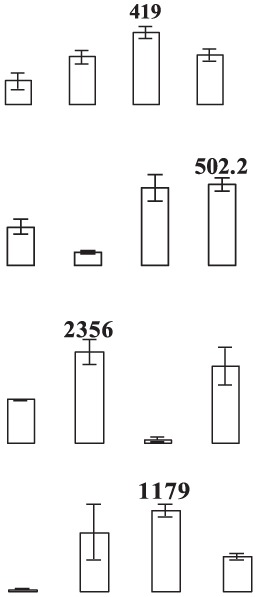	GSMUA_Achr2P16710_001	V-type proton ATPase subunit B2	54.5/4.79	6	66.3
E34		GSMUA_Achr1P22590_001	Putative Protein SEC13 homolog	46.9 /8.67	12	67.7
E47		GSMUA_Achr1P25320_001	Putative Sec14 cytosolic factor	75.9/8.5	12	64
E58		GSMUA_Achr8P08270_001	Putative Protein tolB	71.9/6.26	4	72.8
**TRANSCRIPTION**						
E60	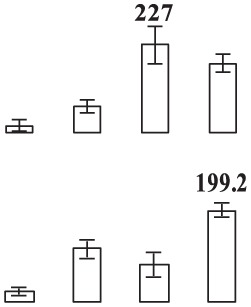	GSMUA_Achr6P04950_001	Putative Far upstream element-binding protein 1	74.9/5.46	9	105
E64		GSMUA_Achr10P07910_001	RNA recognition motif containing protein, putative, expressed	26.8/5.67	5	59.4
**OTHERS**						
E20	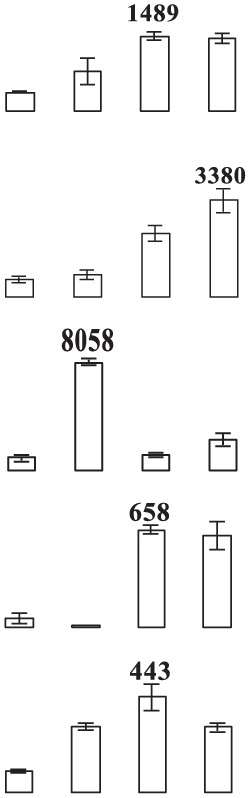	GSMUA_Achr10P03730_001	Actin-2 >ITC1587_Bchr10_P29159|actin	41.8/5.17	18	71.2
E22		GSMUA_Achr6P20120_001	Golgin subfamily B member 1-like isoform X1	218.3/4.45	42	65.7
E94		ITC1587_Bchr5_T13048	|agenet domain containing protein expressed	232.6/4.92	28	63.4
E7		GSMUA_Achr8P12870_001	OSIGBa0147B06.5 protein	25.0/8.71	11	94.1
E14		GSMUA_Achr11P23020_001	Hypothetical protein	11.9/10.8	6	58.8

Protein accumulation is presented using columns, and the accumulation at the control fruit after 1 and 4 days of cold storage, and the ethylene-treated fruit after 1 and 4 days of cold storage are shown from left to right, respectively. The highest gray mean values of the spots in each 2-DE gel samples are presented on the top of the column. Mr, molecular weight; IP, isoelectric point. Protein information came from the banana genome (http://banana-genome.cirad.fr/).

Of the 15 proteins involved in energy metabolism, eight proteins (fructose-bisphospahte aldolase cytoplasmic isozyme, malate dehydrogenase, carboxyvinyl-carboxyphosphonate phosphory-lmutase, fumarate hydratase 1, pyruvate dehydrogenase E1, phosphoglycerate kinase, and RuBisCO large subunit-binding protein subunit alpha) exhibited up-regulation while one protein (ATP synthase 24 kDa subunit) exhibited down-regulation in banana fruit after ethylene treatment during low temperature storage. Three proteins (malate dedycrogenase, aldehyde dehydgrogenase family 2 member B7 and RuBisCO large subunit-binding protein β-subunit) were up-regulated after 1 days of storage, but down-regulated after 4 days of storage. Two other proteins (cytochrome b6-f complex iron-sulfur subunit and thylakoid luminal 15 kDa protein) were down-regulated after 1 days of storage.

Eight stress and defense-associated proteins were up-regulated, including two L-ascorbate peroxidase, one peroxidase, two thaumatin-like proteins, and three heat shock cognate 70 kDa proteins, whereas one was down-regulated by ethylene treatment during low temperature storage. One superoxide dismutase [Cu-Zn]) showed down-regulation after 1 days of storage and up-regulation after 4 days of storage. One peroxiredoxin-2C and one thaumatin-like protein were up-regulated after only 1 days of storage while one heat shock cognate 70 kDa protein was up-regulated after 4 days of storage after ethylene treatment.

The major proteins from the primary metabolism category were methionine salvage cycle-related proteins. All six *S*-adenosylmethionine synthases were up-regulated by ethylene treatment. Two other methionine salvage cycle-related proteins (1,2-dihydroxy- 3-keto-5-methylthiopentene dioxygenase) were up-regulated after 4 days of cold storage in the ethylene treated fruit. In addition, one aminotransferase y4uB and one biotin carboxylase were up-regulated while one acidic endochitinase was down-regulated during cold storage. UTP-glucose-1-phosphate uridylyltransferase, involved in polysaccharide metabolism, was also induced by ethylene.

Eight proteins involved in protein metabolic process were identified. Of these proteins, six proteins (two chaperone protein ClpB2, two peptidyl-prolyl cis-trans isomerase, one cell division protease ftsH homolog 2 and one ATP-dependent Clp protease) showed significant up-regulation by ethylene treatment after 1 days and/or 4 days of cold storage. One eukaryotic translation initiation factor 5A protein showed down-regulation after 1 days of storage and up-regulation after 4 days of storage. One FK506-bindng protein 2 was down-regulated by ethylene treatment during cold storage.

Concerning signal transduction, only two proteins with significant differences were observed, i.e., IAA-amino acid hydrolase and a septum-promoting GTP-binding protein, which were significantly up-regulated by ethylene treatment in the early response to low temperature. Of four transportation-related proteins, three (V-type proton ATPase subunit B2, protein Sec 13 and Sec 14) were up-regulated and one (protein tolB) was down-regulated by ethylene treatment. In addition, two transcription-related proteins, far upstream element-binding protein and RNA recognition motif containing protein, were found to be up-regulated by ethylene treatment.

## Discussion

### Ethylene induces chilling tolerance in harvested banana fruit

Most fruits and vegetables from tropical and subtropical areas are relatively sensitive to low temperature. Banana fruit develop CI symptoms when stored below 12°C (Jiang et al., 2004). In this study, ethylene pre-treatment induced chilling tolerance, with fruit exhibiting no browning and pitting after 4 days of storage at 6°C (Figures 1,2A).

The maximal PSII quantum yield (*Fv/Fm*), a chlorophyll fluorescence parameter, can reflect the changes in energy transfer process and chloroplast activity, which has been widely used for monitoring stress response in plants (Huang et al., 2012). Relative leakage rate is an important index to evaluate cellular membrane integrity. Cold stress leads to a rapid increase in relative leakage rate in some harvested fruits (Aghdam and Bodbodak, 2013). In the present study, compared with control fruit, almost constant *Fv/Fm* and significantly lower relative leakage rates were observed in the ethylene-pretreated fruit after 4 days of cold storage, further confirming that the treatment enhanced chilling tolerance in harvested banana fruit.

### Proteomic changes induced by ethylene treatment in harvested banana fruit at low temperature

The current study focused on the effect of ethylene on cold storage in harvested fruits. However, there is little information on the involvement of ethylene treatment in this biological process with regard to proteomics. The present work performed a comparative proteomic analysis between ethylene-treated and control fruit during cold storage. Among the differentially expressed proteins, the vast majority were up-regulated by ethylene treatment, but a minority of differential expressed proteins were down-regulated.

#### Proteins related to energy metabolism

Energy supply in cellular activity plays an important role in regulating senescence of postharvest fruit (Liu et al., 2007). Inadequate energy supplies or reduced efficiency of cellular energy generation usually results in physiological disorder and skin browning in harvested fruits (Duan et al., 2004; Jin et al., 2014). Perotti et al. (2015) suggested that the increased energy generation from glycolysis might be required to counter cold stress. Some postharvest treatments improved energy status and, thus, induced chilling tolerance in fruits (Carvajal et al., 2015; Jin et al., 2015).

Glycolysis is the metabolic pathway that converts glucose into pyruvate and forms the high-energy compounds, ATP and NADH. In this study, two proteins, one cytosol fructose-bisphosphate aldolase (FBA, E15) and one phosphoglycerate kinase (PGK, E25), involved in glycolysis were significantly up-regulated by ethylene pretreatment in harvested banana fruit stored at 6°C (Table 1, Figure 4). FBA is an enzyme catalyzing the reversible reaction that splits the aldol, fructose 1,6-bisphosphate, into the triose phosphates dihydroxyacetone phosphate and glyceraldehyde 3-phosphate. The increase in the expression of cytosol *FBA* gene and its enzymatic activity contributed to ATP synthesis through the promotion of glycolytic pathway (Konishi et al., 2004). Fan et al. (2009) found that NaCl, ABA, and PEG could trigger a significant induction of *SpFBA* in *Sesuvium portulacastrum* root and overproduction of recombinant SpFBA resulted in an increased tolerance to salinity in transgenic *Escherichia coli*. Similarly, two FBA proteins were significantly induced by mild osmotic stress in drought tolerant banana AAB variety (Vanhove et al., 2012). PGK catalyzes the reversible transfer of a phosphate group from 1,3-bisphosphoglycerate (1,3-BPG) to ADP, producing 3-phosphoglycerate (3-PG) and ATP. PGK acts in the first ATP-generating step of the glycolytic pathway. Liu et al. (2015) found that the expression of *AtPGK2* could be induced significantly by salt stress and over-expression of AtPGK2 conferred salt tolerance in transgenic *Arabidopsis* plants. Carpentier et al. (2007) also demonstrated that up-regulated PGKs were associated with dehydration tolerance in banana shoot meristem. It suggests that the induced expression of FBA and PGK by ethylene contributed to ATP synthesis, which was important for banana fruit to deal with oxidative stress.

Tricarboxylic acid (TCA) cycle-related proteins are another group of major up-regulated proteins by ethylene pretreatment in banana fruit stored at 6°C. In this subgroup, three malate dehydrogenases (MDH) were up-regulated by ethylene pretreatment, but showed differential expression patterns. Of the three MDH proteins, two MDHs (E37 and E92) were up-regulated by ethylene pretreatment only after 4 days of cold storage at 6°C (Figure 4, Table 1) while the third MDH showed up-regulation after 1 days of storage but down-regulation after 4 days of storage in the ethylene-treated fruit as compared to control fruits. MDH reversibly catalyzes the oxidation of malate to oxaloacetate with energy release via NADH. There were some reports with regard to the involvement of MDH in abiotic stress. Yao et al. (2011) observed that the expression of *MdcyMDH* gene was strongly down-regulated in apple subjected to chilling injury and the over-expression of *MdcyMDH* enhanced cold and salt tolerance, which might be related to altered ATP generation. Faghani et al. (2015) and Sánchez-Bel et al. (2012a) reported that MDHs were involved in drought stress in wheat and chilling stress in bell pepper fruit, respectively. Pyruvate dehydrogenase E1 is the first component enzyme of pyruvate dehydrogenase complex (PDC). Pyruvate dehydrogenase links the glycolytic pathway to the citric acid cycle which releases energy via NADH. In this study, one pyruvate dehydrogenase (E13) was up-regulated by ethylene pretreatment in banana fruit during storage (Figure 4). However, no direct evidence has been reported that pyruvate dehydrogenase is involved in abiotic stress in plants. Thus, the results suggest that the up-regulated MDH and pyruvate dehydrogenase were beneficial for banana fruit by releasing energy to cope with the cold stress.

#### Proteins related to stress response and defense

Like other biotic and abiotic stresses, abnormal low temperature results in the accumulation of reactive oxygen species (ROS) due to the disruption of cellular redox homeostasis, which cause oxidative damage of biomacromolecules such as protein, lipid and DNA. To cope with oxidative stress, plants have developed a complex antioxidant system to protect against oxidative damage, including antioxidant enzymes and low molecular mass antioxidants. The important antioxidant enzymes consist of superoxide dismutase (SOD), catalase (CAT), ascorbate peroxidase (APX), glutathione peroxidase (GPX), glutathione-S-transferase (GST), monodehydroascorbate reductase (MDHAR), dehydroascorbate reductase (DHAR), and gluthatione reductase (GR) (Moller, 2001). In addition, 2-Cys peroxiredoxin (2-Cys Prx) as a member of peroxidase superfamily plays an important role in detoxification of hydrogen peroxide, alkylhydroperoxides and peroxynitrites in plants (Dietz, 2011). In the present study, two APXs (E10, E68) and one peroxidase (E69) were significantly up-regulated by ethylene pretreatment during the early and late cold stress condition (Table 1, Figure 4). However, one 2-Cys Prx and one SOD was up-regulated by ethylene pretreatment at the early and late low temperature stage, respectively. Many studies have shown that CAT, peroxidase and SOD could have a major influences on the degree of sensitivity or tolerance to low temperature stress (Sevillano et al., 2009). Sato et al. (2001) suggested that APX activity induced by heat treatment is a key element in protecting rice against exposure to low temperature. It is also noted that *Arabidopsis* mutants lacking chloroplastid 2-Cys-Prx increased H_2_O_2_ levels and altered redox homeostasis in leaves (Pulido et al., 2010). Our results indicated that APX, peroxidase, SOD and 2-Cys Prx in banana fruit at different storage stages were up-regulated by ethylene pretreatment, which coordinately improved the chilling tolerance in harvested banana fruit.

Heat shock proteins (Hsps) are stress-responsive family of proteins with molecular weights ranging from 15 to 115 kDa. Of these Hsps, five families have been identified, including Hsp70, Hsp60, Hsp90, Hsp100, and small Hsp (sHsp). Hsps exert their protective effect against various stresses mainly due to their chaperone activity. Some studies reported that Hsps induced by various postharvest treatments such as heat treatment, and salicylate and jasmonate treatments, are involved in the chilling tolerance in fruits (Aghdam et al., 2013), suggesting a central role of Hsps in acquired tolerance to chilling stress. Heat shock congnate 70 kDa protein (Hsc70) is a member of heat shock protein 70 (Hsp70) family, which is located in both the nuclear and cytoplasmic compartments and plays a role in protein folding and translocation (Kaur et al., 2013). Unlike canonical heat shock proteins, Hsc70 is constitutively expressed and is related to normal cellular process. It is reported that Hsc70 might confer abiotic stress tolerance to *Pennisetum glaucum* (Reddy et al., 2010) and *Arabidopsis thaliana* (Cazalé et al., 2009). In this study, four spots (E5, E63, E66 and E91) were identified as the same Hsc70 protein (GSMUA_Achr8P20830_001), possibly due to posttranslational modification of the protein. The four Hsc70 proteins were significantly up-regulated by ethylene pretreatment in banana fruit under cold stress, but showing differential expressions patterns. Two Hsc70 proteins (E5, E91) were up-regulated after 1 and 4 days of cold storage. Hsc70 protein (E63) was up-regulated after 1 days of storage while Hsc70 (E91) was up-regulated after 4 days of storage by ethylene pretreatment. Similarly, Carpentier et al. (2007) reported that the same trail of 6 spots was identified as HSP70-like in banana meristem. Vanhove et al. (2015) further confirmed that chromosome location of the 6 spots and then found that only spot 5 was more responsive to osmotic stress and significantly up-regulated by osmotic stress. The study suggested that different Hsc70s might play a role in ethylene-induced chilling tolerance in harvested banana fruit at different stages of low temperature storage.

Thaumatin-like proteins (TLPs) are a class of pathogenesis related proteins, which are induced in response to pathogens. Over-expression of TLPs in plants enhanced the resistance to biotic stress (Acharya et al., 2013). Our study found that three TLPs were greatly induced by ethylene pretreatment, especially when banana fruit were stored for 1 day at 6°C (Table 1 and Figure 4). Dagar et al. (2010) also observed that the expression of thaumatin-like protein 1 precursors were significantly higher in the chilling injury-resistant peach than in the susceptible cultivar. Therefore, it is suggested that thaumatin-like protein might be involved in the induced-chilling tolerance in harvested banana fruit, but the mechanism needs further investigation.

#### Proteins related to primary metabolism

In this study, the detected proteins from primary metabolism were mainly methionine salvage cycle-related proteins. *S*-adenosylmethionine synthase (SAMS) catalyzes the synthesis of *S*-adenosylmethionine (SAM) from ATP and L-methionine. SAM is the major methyl group donor and its synthesis is involved in numerous transmethylations of secondary metabolites and macromolecules in plants (Sauter et al., 2013). More importantly, SAM serves as a precursor of polyamine and ethylene, which are important regulators in fruit ripening, senescence and response to abiotic stress (Sauter et al., 2013). Our study showed that all six differentially expressed *S*-adenosylmethionine synthases were up-regulated in the ethylene-pretreated banana fruit in comparison to the control fruit under cold stress conditions, implying that *S*-adenosylmethionine synthases play an important role in ethylene-induced chilling tolerance. Of these six differentially expressed SAMs, E23 and E54 spots, E33 and E87 spots, were identified as the same proteins: GSMUA_Achr2 P07970 and GSMUA_Achr5 P20990, respectively. Five SAMSs protein (E23, E54, E33, E87, and E36) were up-regulated after 1 and 4 days of cold storage while one SAM (E18) was up-regulated after only 1 days of storage by ethylene pretreatment. Recently, Guo et al. (2014) reported that ABA, H_2_O_2_ and NO interactions mediated the cold-induced *MfSAMS1* expression and cold acclimation in *Medicago sativa* subsp, and suggested that SAM increased the cold tolerance via up-regulating polyamine oxidation. However, a SAM protein was significantly down-regulated by mild osmotic stress in drought tolerant banana ABB variety (Vanhove et al., 2012), implying that SAMs play different roles under abiotic stress condition. In addition, two other methionine salvage cycle-related proteins, 1,2-dihydroxy-3-keto-5-methylthiopentene dioxygenases (MTCBP-1s), were up-regulated in the late development of cold tolerance. MTCBP-1 catalyzes the formation of formate and 2-keto-4-methylthiobutyrate (KMTB) from 1,2-dihydroxy-3-keto-5-methylthiopentene (DHK-MTPene), providing the substrate for the synthesis of methionine, in the methionine salvage pathway (Sauter et al., 2013). However, there are no reports on the involvement of MTCBP-1 in plant tolerance to abiotic stress. It is suggested that the up-regulated SAM and MTCBP-1 might contribute to polyamines biosynthesis and cold tolerance of banana fruit.

#### Proteins related to protein metabolism

Abiotic stresses usually lead to protein denaturation and dysfunction. Plants possess some mechanisms to cope with the adverse situation, including protein refolding and protein degradation. In the present study, four refolding-related proteins [two chaperone ClpB2 proteins (E2, E61) and two peptidyl-prolyl cis-trans isomerases CYP20-2 proteins (E86, E95)] and two protein degrading-related proteins (FtsH and ClpP, E1 and E3) were significantly up-regulated by ethylene pretreatment in banana fruit.

It is well known that living organisms respond to different stress conditions with the induction of heat shock proteins (Hsps), which exert the protective effect against these stresses by refolding damaged proteins, preventing protein aggregation and renaturing aggregated proteins. The Casein lytic proteinase/heat shock protein 100 (Clp/Hsp100) proteins are chaperones that act to remodel/disassemble protein complexes using ATP. In the present study, two chaperone ClpB2 proteins were significantly up-regulated by ethylene pretreatment in banana fruit after 1 days of cold storage without CI symptom. Peptidyl-prolyl cis/trans isomerases (PPIases) represent an important type of protein foldase that can accelerate the energetically unfavorable cis-trans isomerization of the peptide bond of proline. Four structurally distinct subfamilies of PPIases (cyclophilins, FK506-binding proteins, parvulins, and PP2A phosphatase activators) have been characterized (Lu et al., 2007). Our results showed that two CYP20-2 proteins belonging to cyclophilins subfamily were up-regulated by ethylene pretreatment. Recently, Lee et al. (2015) reported that the OsSKIP-OsCYP18-2 interaction played an important role in the transcriptional and post-transcriptional regulation of the stress-related genes and increased the tolerance to drought stress. Kumari et al. (2013) reviewed that some members of cyclophilin subfamily were directly linked to multiple stresses. Therefore, the induced expression of ClpB2 and CYP20-2 were thought to participate in the early and late response to cold stress, respectively.

Protein degradation plays an important role in maintaining cellular process by removing misfolded or damaged proteins and controlling the level of certain regulatory. Caseinolytic protease (ClpP) is an energy-dependent serine protease which unfolds protein substrates and translocates them into the degradation chamber of ClpP through a central pore. The thylakoid membrane-anchored ATP-dependent protease FtsH is involved in the processive degradation of stroma-exposed thylakoid proteins (Malnoe et al., 2014). ClpP and FtsH play vital role in the protein quality control of mitochondrial matrix and thylakoid membrane, respectively. Sanchez-Bel et al. (2012b) observed that two proteins (26S proteasome subunit RPN11 and aspartic proteinase) participating in the protein degradation were down-regulated in tomato in response to cold stress. There are no reports on the link between the activities of ClpP and FtsH and tolerance to cold stress conditions. Given the significantly up-regulated expression of ClpP and FtsH by ethylene pretreatment under cold stress, it is suggested that ClpP and FtsH played an important role in ethylene-induced chilling tolerance.

Environment stress leads to the global reduction of protein synthesis, while the translation of selected stress responsive proteins can be maintained (Bavli-Kertselli et al., 2015). Translation regulation is always assisted by eukaryotic initiation factors (eIFs) (Xu, 2011). Initiation factor 5A (eIF5A) is a small protein involved in the formation of the first peptide bond. Recently, it has been shown that eIF05A is involved in abiotic stress tolerance in plants. Xu (2011) found that over-expressing RceIF05A of transgenic *Arabidopsis* improved the resistance to heat, oxidative and osmotic stresses, while the plant with reduced eIF5A expression was more susceptible to these stresses. Wang et al. (2012) reported that transgenic yeast and poplar expressing TaeIF5A1 elevated protein levels combined with improved abiotic stresses tolerance. In the study, control fruit with down-regulated eIF5A showed the appearance of severe chilling injury, while the protein was significantly up-regulated in the ethylene- pretreated fruit without any chilling injury symptom.

#### Proteins related to signal transduction, transport, and transcription

Under abiotic or biotic stress conditions, plants typically respond by sensing and transferring stress signals through signal transduction networks. It was reported that ethylene signal transduction elements were involved in chilling injury in non-climacteric loquat fruit (Wang et al., 2010). Unfortunately, in this study, no ethylene signal transduction elements were identified, possibly resulting from the limited 2 DE detection parameters (pH 5–8 and molecular weight of 15–100 kDa). However, two other signal transduction-related proteins, IAA-amino acid hydrolase ILR1-like 1 (E82), and Septum-promoting GTP-binding protein 1 (E16), were greatly up-regulated by ethylene pretreatment after 4 days of cold storage. IAA-amino acid hydrolase ILR1 initially isolated from *Arabidopsis thaliana* releases active IAA from conjugates through cleaving IAA-amino acid conjugates (Campanella et al., 2003). Agami and Mohamed (2013) showed that IAA pretreatment alleviated cadmium-toxicity in wheat seedlings through enhancing the activities of antioxidant enzymes. Zhou et al. (2014) also reported that IAA-amino acid hydrolase ILR1might be involved in boron-induced alleviation of aluminum-toxicity. The up-regulated IAA-amino acid hydrolase ILR1 by ethylene pretreatment might contribute to enhancement of free IAA level.

Vacuole H+-ATPase (VHA) is one of plant proton pumps in the tonoplast. VHA and vacuole H+-pyrophosphatase create the proton gradient and membrane potential and perform the transport of ions and metabolites across the tonoplast by hydrolyzing ATP. Some studies have shown that VHA plays a role in enhancing stress tolerance in plants. Dong et al. (2013) reported that over-expression of apple VHA-A gene conferred am increase in the drought tolerance of transgenic tobacco seedlings with enhanced vacuole H+-ATPase (VHA) activity. Sanchez-Bel et al. (2012b) also found that V-ATPase was inhibited in chilled tomato fruit. In this study, VHA (E70) was significantly up-regulated in the ethylene-pretreated fruit after 1 day of cold storage at 6°C, compared with control fruit, suggesting that V-ATPase might be involved in the early response to cold stress by regulating membrane trafficking.

Two transcription-related proteins which were found to be up-regulated by ethylene treatment are RNA recognition motif containing protein (E64) and far upstream element-binding protein (E60). RNA-binding proteins (RBPs) play roles in transcription, RNA processing, localization and stability, and translation (Shi et al., 2015). RNA recognition motif (RRM) is one of the most abundant RNA binding domains (Maris and Dominguez, 2005). It has been reported that RBPs contribute to enhancing cold and freezing tolerance (Kim et al., 2005). However, there is no information on the involvement of far upstream element-binding protein in the response to abiotic stress. It is suggested that the up-regulated transcription-related proteins are beneficial to the transcription or translation of some stress-responsive genes.

## Conclusion

In this study, differentially expressed proteins by ethylene pretreatment in banana fruit under cold stress condition are mainly linked to energy metabolism, stress response and defense, and methionine salvage cycle. It is suggested that the up-regulated energy metabolism-related proteins contribute to providing sufficient ATP for lipid synthesis, protein refolding and protein degradation. Most of the differentially expressed proteins associated with stress response are involved in ROS clearance and protein protection. Surprisingly, multiple SAM synthesis-related proteins were up-regulated, which is possibly beneficial to the biosynthesis of polyamine and enhancing chilling tolerance. A widespread up-regulation of proteins in abundance indicates that protein induction by ethylene treatment seems to be essential to increasing chilling injury tolerance in harvested banana fruit. The identification of novel ethylene-regulated proteins provides not only new insights into chilling stress responses of banana fruit but also a new clue for further dissection of their functions.

## Author contributions

TL and XD conceived and designed the study. TL, ZY, and DZ performed the experiments and analyze the data. TL and XD drafted the manuscript. All authors participated in the interpretation of data, and the revision of the manuscript. All authors approved the submission and agreed to be accountable for all aspects of the work.

### Conflict of interest statement

The authors declare that the research was conducted in the absence of any commercial or financial relationships that could be construed as a potential conflict of interest.

## References

[B1] Acharya K. Pal A. K. Gulati A. Kumar S. Singh A. K. Ahuja P. S. (2013). Overexpression of camellia sinensis thaumatin-Like protein, CsTLP in potato confers enhanced resistance to *Macrophomina phaseolina* and *Phytophthora infestans* Infection. Mol. Biotechnol. 54, 609–622. 10.1007/s12033-012-9603-y 23086453

[B2] Agami R. A. Mohamed G. F. (2013). Exogenous treatment with indole-3-acetic acid and salicylic acid alleviates cadmium toxicity in wheat seedlings. Ecotox. Environ. Safe 94, 164–171. 10.1016/j.ecoenv.2013.04.013 23684274

[B3] Aghdam M. S. Bodbodak S. (2013). Physiological and biochemical mechanisms regulating chilling tolerance in fruits and vegetables under postharvest salicylates and jasmonates treatments. Sci. Hortic. 156, 73–85. 10.1016/j.scienta.2013.03.028

[B4] Aghdam M. S. Sevillano L. Flores F. B. Bodbodak S. (2013). Heat shock proteins as biochemical markers for postharvest chilling stress in fruits and vegetables. Sci. Hortic. 160, 54–64. 10.1016/j.scienta.2013.05.020

[B5] Alexander L. Grierson D. (2002). Ethylene biosynthesis and action in tomato: a model for climacteric fruit ripeing. J. Exp. Bot. 53, 2039–2055. 10.1093/jxb/erp072 12324528

[B6] Bavli-Kertselli I. Melamed D. Bar-Ziv L. Volf H. Arava Y. (2015). Overexpression of eukaryotic initiation factor 5 rescues the translational defect of tpk1 w in a manner that necessitates a novel phosphorylation site. FEBS J. 282, 504–520. 10.1111/febs.13158 25417541

[B7] Campanella J. J. Ludwig-Mueller J. Bakllamaja V. Sharma V. Cartier A. (2003). ILR1 and sILR1 IAA amidohydrolase homologs differ in expression pattern and substrate specificity. Plant Growth Regul. 41, 215–223. 10.1023/B:GROW.0000007501.27878.aa

[B8] Candan A. P. Graell J. Larrigaudiere C. (2011). Postharvest quality and chilling injury of plums: benefits of 1-methylcyclopropene. Span. J. Agric. Res. 9, 554–564. 10.1016/j.agsy.2011.02.001

[B9] Cao S. F. Zheng Y. H. Wang K. T. Rui H. J. Shang H. T. Tang S. S. (2010). The effects of 1-methylcyclopropene on chilling and cell wall metabolism in loquat fruit. J. Hortic. Sci. Biotechnol. 85, 147–153.

[B10] Carpentier S. C. Witters E. Laukens K. Van Onckelen H. Swennen R. Panis B. (2007). Banana (Musa spp.) as a model to study the meristem proteome: acclimation to osmotic stress. Proteomics 7, 92–105. 10.1002/pmic.200600533 17149779

[B11] Carvajal F. Palma F. Jamilena M. Garrido D. (2015). Preconditioning treatment induces chilling tolerance in zucchini fruit improving different physiological mechanisms against cold injury. Ann. Appl. Biol. 166, 340–354. 10.1111/aab.12189

[B12] Cazalé A. C. Clément M. Chiarenza S. Roncato M. A. Pochon N. Creff A.. (2009). Altered expression of cytosolic/nuclear HSC70-1 molecular chaperone affects development and abiotic stress tolerance in *Arabidopsis thaliana*. J. Exp. Bot. 60, 2653–2664. 10.1093/jxb/erp109 19443614

[B13] Dagar A. Friedman H. Lurie S. (2010). Thaumatin-like proteins and their possible role in protection against chilling injury in peach fruit. Postharvest Biol. Tec. 57, 77–85. 10.1016/j.postharvbio.2010.03.009

[B14] Dietz K. J. (2011). Peroxiredoxins in plants and cyanobacteria. Antioxid. Redox Signal. 15, 1129–1159. 10.1089/ars.2010.3657 21194355 PMC3135184

[B15] Dong L. Zhou H. W. Sonego L. Lers A. Lurie S. (2001). Ethylene involvement in the cold storage disorder of ‘Flavortop’ nectarine. Postharvest Biol. Tec. 23, 105–115. 10.1016/S0925-5214(01)00106-5

[B16] Dong Q. L. Wang C. R. Liu D. D. Hu D. G. Fang M. J. You C. X.. (2013). MdVHA-A encodes an apple subunit A of vacuolar H+-ATPase and enhances drought tolerance in transgenic tobacco seedlings. J. Plant Physiol. 170, 601–609. 10.1016/j.jplph.2012.12.014 23399407

[B17] Duan X. W. Jiang Y. M. Su X. G. Liu H. Li Y. B. Zhang Z. Q. (2004). Role of pure oxygen treatment in browning of litchi fruit after harvest. Plant Sci. 167, 665–668. 10.1016/j.plantsci.2004.05.009

[B18] Faghani E. Gharechahi J. Komatsu S. Mirzaei M. Khavarinejad R. A. Najafi F.. (2015). Comparative physiology and proteomic analysis of two wheat genotypes contrasting in drought tolerance. J. Proteom. 114, 1–15. 10.1016/j.jprot.2014.10.018 25449836

[B19] Fan W. Zhang Z. Zhang Y. (2009). Cloning and molecular characterization of fructose-1,6-bisphosphate aldolase gene regulated by high-salinity and drought in *Sesuvium portulacastrum*. Plant Cell Rep. 28, 975–984. 10.1007/s00299-009-0702-6 19381641

[B20] Fan X. Argenta L. Mattheis J. P. (2002). Interactive effects of 1-MCP and temperature on ‘Elberta’ peach quality. Hortscience 37, 134–138.

[B21] Fan X. T. Mattheis J. P. (1999). Development of apple superficial scald, soft scald, core flush, and greasiness is reduced by MCP. J. Agr. Food Chem. 47, 3063–3068. 10.1021/jf981176b 10552609

[B22] Guo Z. Tan J. Zhuo C. Wang C. Xiang B. Wang Z. (2014). Abscisic acid, H_2_O_2_ and nitric oxide interactions mediated cold-induced S-adenosylmethionine synthetase in *Medicago sativa* subsp falcata that confers cold tolerance through up-regulating polyamine oxidation. Plant Biotechnol. J. 12, 601–612. 10.1111/pbi.12166 24517136

[B23] Huang S. Li T. Jiang G. Xie W. Chang S. Jiang Y. (2012). 1-Methylcyclopropene reduces chilling injury of harvested okra (*Hibiscus esculentus* L.) pods. Sci. Hortic. 141, 42–46. 10.1016/j.scienta.2012.04.016

[B24] Jiang Y. M. Joyce D. C. Jiang W. B. Lu W. J. (2004). Effects of chilling temperatures on ethylene binding by banana fruit. Plant Growth Regul. 43, 109–115. 10.1023/B:GROW.0000040112.19837.5f

[B25] Jin P. Zhang Y. Shan T. Huang Y. Xu J. Zheng Y. (2015). Low-temperature conditioning alleviates chilling injury in loquat fruit and regulates glycine betaine content and energy status. J. Agr. Food Chem. 63, 3654–3659. 10.1021/acs.jafc.5b00605 25822129

[B26] Jin P. Zhu H. Wang L. Shan T. Zheng Y. (2014). Oxalic acid alleviates chilling injury in peach fruit by regulating energy metabolism and fatty acid contents. Food Chem. 161, 87–93. 10.1016/j.foodchem.2014.03.103 24837925

[B27] Kaur G. Lieu K. G. Jans D. A. (2013). 70-kDa heat shock cognate protein hsc70 mediates calmodulin-dependent nuclear import of the sex-determining factor SRY. J. Biol. Chem. 288, 4148–4157. 10.1074/jbc.M112.436741 23235156 PMC3567665

[B28] Kim Y. O. Kim J. S. Kang H. (2005). Cold-inducible zinc finger-containing glycine-rich RNA-binding protein contributes to the enhancement of freezing tolerance in *Arabidopsis thaliana*. Plant J. 42, 890–900. 10.1111/j.1365-313X.2005.02420.x 15941401

[B29] Konishi H. Yamane H. Maeshima M. Komatsu S. (2004). Characterization of fructose-bisphosphate aldolase regulated by gibberellin in roots of rice seedling. Plant Mol. Biol. 56, 839–848. 10.1007/s11103-004-5920-2 15821984

[B30] Kumari S. Roy S. Singh P. Singla-Pareek S. L. Pareek A. (2013). Cyclophilins: proteins in search of function. Plant Signal. Behav. 8, e22734–e22734. 10.4161/psb.22734 23123451 PMC3745578

[B31] Lee S. S. Park H. J. Yoon D. H. Kim B. G. Ahn J. C. Luan S.. (2015). Rice cyclophilin OsCYP18-2 is translocated to the nucleus by an interaction with SKIP and enhances drought tolerance in rice and Arabidopsis. Plant Cell Environ. 38, 2071–2087. 10.1111/pce.12531 25847193

[B32] Lelievre J. M. Latche A. Jones B. Bouzayen M. Pech J. C. (2007). Ethylene and fruit ripening. Physiol. Plantarum. 101, 727–739. 10.1034/j.1399-3054.1997.1010408.x

[B33] Lin Z. Zhong S. Grierson D. (2009). Recent advances in ethylene research. J. Exp. Bot. 60, 3311–3336. 10.1093/jxb/erp204 19567479

[B34] Liu D. Li W. C. Cheng J. F. Hou L. (2015). AtPGK2, a member of PGKs gene family in Arabidopsis, has a positive role in salt stresstolerance. Plant Cell Tiss. Org. 120, 251–262. 10.1007/s11240-014-0601-6

[B35] Liu H. Song L. Jiang Y. Joyce D. C. Zhao M. You Y. (2007). Short-term anoxia treatment maintains tissue energy levels and membrane integrity and inhibits browning of harvested litchi fruit. J. Sci. Food Agr. 87, 1767–1771. 10.1002/jsfa.2920

[B36] Lu K. P. Finn G. Lee T. H. Nicholson L. K. (2007). Prolyl cis-trans isomerization as a molecular timer. Nat. Chem. Biol. 3, 619–629. 10.1038/nchembio.2007.35 17876319

[B37] Ma Q. Suo J. Huber D. J. Dong X. Han Y. Zhang Z. (2014). Effect of hot water treatments on chilling injury and expression of a new C-repeat binding factor (CBF) in ‘Hongyang’ kiwifruit during low temperature storage. Postharvest Biol. Tec. 97, 102–110. 10.1016/j.postharvbio.2014.05.018

[B38] Malnoe A. Wang F. Girard-Bascou J. Wollman F. A. de Vitry C. (2014). Thylakoid FtsH protease contributes to photosystem II and cytochrome b(6)f remodeling in chlamydomonas reinhardtii under stress conditions. Plant Cell 26, 373–390. 10.1105/tpc.113.120113 24449688 PMC3963582

[B39] Maris C. Dominguez C. (2005). Allain FHT: the RNA recognition motif, a plastic RNA-binding platform to regulate post-transcriptional gene expression. FEBS J. 272, 2118–2131. 10.1111/j.1742-4658.2005.04653.x 15853797

[B40] Moller I. M. (2001). Plant mitochondria and oxidative stress: electron transport, NADPH turnover, and metabolism of reactive oxygen species. Annu. Rev. Plant Phys. Plant Mol. Biol. 52, 561–591. 10.1146/annurev.arplant.52.1.561 11337409

[B41] Perotti V. E. Moreno A. S. Trípodi K. E. Meier G. Bello F. Cocco M.. (2015). Proteomic and metabolomic profiling of Valencia orange fruit after natural frost exposure. Physiol. Plantarum. 153, 337–354. 10.1111/ppl.12259 25132553

[B42] Pesis E. Ackerman M. Ben-Arie R. Feygenberg O. Feng X. Q. Apelbaum A. (2002). Ethylene involvement in chilling injury symptoms of avocado during cold storage. Postharvest Biol. Tec. 24, 171–181. 10.1016/S0925-5214(01)00134-X

[B43] Porat R. Weiss B. Cohen L. Daus A. Goren R. Droby S. (1999). Effects of ethylene and 1-methylcyclopropene on the postharvest qualities of ‘Shamouti’ oranges. Postharvest Biol. Tec. 15, 155–163. 10.1016/S0925-5214(98)00079-9

[B44] Pulido P. Spínola M. C. Kirchsteiger K. Guinea M. Belen Pascual M. Sahrawy M.. (2010). Functional analysis of the pathways for 2-Cys peroxiredoxin reduction in *Arabidopsis thaliana* chloroplasts. J. Exp. Bot. 61, 4043–4054. 10.1093/jxb/erq218 20616155 PMC2935875

[B45] Reddy P. S. Mallikarjuna G. Kaul T. Chakradhar T. Mishra R. N. Sopory S. K.. (2010). Molecular cloning and characterization of gene encoding for cytoplasmic Hsc70 from *Pennisetum glaucum* may play a protective role against abiotic stresses. Mol. Genet. Genomics 283, 243–254. 10.1007/s00438-010-0518-7 20127116

[B46] Sanchez-Bel P. Egea I. Sanchez-Ballesta M. T. Sevillano L. Del Carmen Bolarin M. Flores F. B. (2012b). Proteome changes in tomato fruits prior to visible symptoms of chilling injury are linked to defensive mechanisms, uncoupling of photosynthetic processes and protein degradation machinery. Plant Cell Physiol. 53, 470–484. 10.1093/pcp/pcr191 22227396

[B47] Sánchez-Bel P. Egea I. Sánchez-Ballesta M. T. Martinez-Madrid C. Fernandez-Garcia N. Romojaro F.. (2012a). Understanding the mechanisms of chilling injury in bell pepper fruits using the proteomic approach. J. Proteom. 75, 5463–5478. 10.1016/j.jprot.2012.06.029 22796354

[B48] Sato Y. Murakami T. Funatsuki H. Matsuba S. Saruyama H. Tanida M. (2001). Heat shock-mediated APX gene expression and protection against chilling injury in rice seedlings. J. Exp. Bot. 52, 145–151. 10.1093/jexbot/52.354.145 11181723

[B49] Sauter M. Moffatt B. Saechao M. C. Hell R. Wirtz M. (2013). Methionine salvage and S-adenosylmethionine: essential links between sulfur, ethylene and polyamine biosynthesis. Biochem. J. 451, 145–154. 10.1042/BJ20121744 23535167

[B50] Selvarajah S. Bauchot A. D. John P. (2001). Internal browning in cold-stored pineapples is suppressed by a postharvest application of 1-methylcyclopropene. Postharvest Biol. Tec. 23, 167–170. 10.1016/S0925-5214(01)00099-0

[B51] Sevillano L. Sanchez-Ballesta M. T. Romojaro F. Flores F. B. (2009). Physiological, hormonal and molecular mechanisms regulating chilling injury in horticultural species. Postharvest technologies applied to reduce its impact. J. Sci. Food Agr. 89, 555–573. 10.1002/jsfa.3468

[B52] Shi X. Hanson M. R. Bentolila S. (2015). Two RNA recognition motif-containing proteins are plant mitochondrial editing factors. Nucleic Acids Res. 43, 3814–3825. 10.1093/nar/gkv245 25800738 PMC4402546

[B53] Toledo T. T. Nogueira S. B. Cordenunsi B. R. Gozzo F. C. Pilau E. J. Lajolo F. M. (2012). Proteomic analysis of banana fruit reveals proteins that are differentially accumulated during ripening. Postharvest Biol. Tec. 70, 51–58. 10.1016/j.postharvbio.2012.04.005

[B54] Vanhove A. C. Vermaelen W. Panis B. Swennen R. Carpentier S. C. (2012). Screening the banana biodiversity for drought tolerance: can an *in vitro* growth model and proteomics be used as a tool to discover tolerant varieties and understand homeostasis. Front Plant Sci. 3:176. 10.3389/fpls.2012.00176 22876254 PMC3410380

[B55] Vanhove A. C. Vermaelen W. Swennen R. Carpentier S. C. (2015). A look behind the screens: characterization of the HSP70 family during osmotic stress in a non-model crop. J. Proteom. 119, 10–20. 10.1016/j.jprot.2015.01.014 25661040

[B56] Vega-Garcia M. O. Lopez-Espinoza G. Chavez O. J. Caro-Corrales J. J. Delgado V. F. Lopez-Valenzuela J. A. (2010). Changes in protein expression associated with chilling injury in tomato fruit. J. Am. Soc. Hortic. Sci. 135, 83–89.

[B57] Wang L. Xu C. Wang C. Wang Y. (2012). Characterization of a eukaryotic translation initiation factor 5A homolog from *Tamarix androssowii* involved in plant abiotic stress tolerance. BMC Plant Biol. 12:118. 10.1186/1471-2229-12-118 22834699 PMC3479025

[B58] Wang P. Zhang B. Li X. Xu C. Yin X. Shan L.. (2010). Ethylene signal transduction elements involved in chilling injury in non-climacteric loquat fruit. J. Exp. Bot. 61, 179–190. 10.1093/jxb/erp302 19884229 PMC2791125

[B59] Wang Y. Luo Z. Du R. Liu Y. Ying T. Mao L. (2013). Effect of nitric oxide on antioxidative response and proline metabolism in banana during cold storage. J. Agr. Food Chem. 6, 8880–8887. 10.1021/jf401447y 23952496

[B60] Wang Y. Luo Z. Huang X. Yang K. Gao S. Du R. (2014). Effect of exogenous γ-aminobutyric acid (GABA) treatment on chilling injury and antioxidant capacity in banana peel. Sci. Hortic. 168, 132–137. 10.1016/j.scienta.2014.01.022

[B61] Xu J. (2011). RceIF5A, encoding an eukaryotic translation initiation factor 5A in Rosa chinensis, can enhance thermotolerance, oxidative and osmotic stress resistance of *Arabidopsis thaliana*. Plant Mol. Biol. 75, 167–178. 10.1007/s11103-010-9716-2 21107886

[B62] Yao Y. X. Li M. Zhai H. You C. X. Hao Y. J. (2011). Isolation and characterization of an apple cytosolic malate dehydrogenase gene reveal its function in malate synthesis. J. Plant Physiol. 168, 474–480. 10.1016/j.jplph.2010.08.008 20934777

[B63] Yun Z. Li W. Pan Z. Xu J. Cheng Y. Deng X. (2010). Comparative proteomics analysis of differentially accumulated proteins in juice sacs of ponkan (*Citrus reticulata*) fruit during postharvest cold storage. Postharvest Biol. Tec. 56, 189–201. 10.1016/j.postharvbio.2010.01.002

[B64] Zhou P. Yang F. Ren X. Huang B. An Y. (2014). Phytotoxicity of aluminum on root growth and indole-3-acetic acid accumulation and transport in alfalfa roots. Environ. Exp. Bot. 104, 1–8. 10.1016/j.envexpbot.2014.02.018

