# Supplementary material for: Proteomic analysis of differentially expressed proteins involved in ethylene-induced chilling tolerance in harvested banana fruit
Source: Front Plant Sci. 2015 Oct 15;6:845. doi: 10.3389/fpls.2015.00845 (PMC4606070; doi:10.3389/fpls.2015.00845)
Supplement: Supplementary file 2 [file DataSheet1.DOC]

SUPPLEMENTAL FIGURE LEGENDS

**Figure S1. The diagrammatic workflow showing the replicates and treatments.** C1, C4, E1 and E4 stood for control fruit at 1 d, control fruit at 4 d, ethylene-treated fruit at 1 d and ethylene-treated fruit at 4 d, respectively. SubG was the abbreviation of subgroup and represented one replicate.

**FIGURE S2. The two-dimensional electrophoresis maps from different replicates.** C1, C4, E1 and E4 stood for control fruit at 1 d, control fruit at 4 d, ethylene-treated fruit at 1 d and ethylene-treated fruit at 4 d, respectively.
