# Supplementary figures and images for: Proteomic analysis of differentially expressed proteins involved in ethylene-induced chilling tolerance in harvested banana fruit
Source: Front Plant Sci. 2015 Oct 15;6:845. doi: 10.3389/fpls.2015.00845 (PMC4606070; doi:10.3389/fpls.2015.00845)

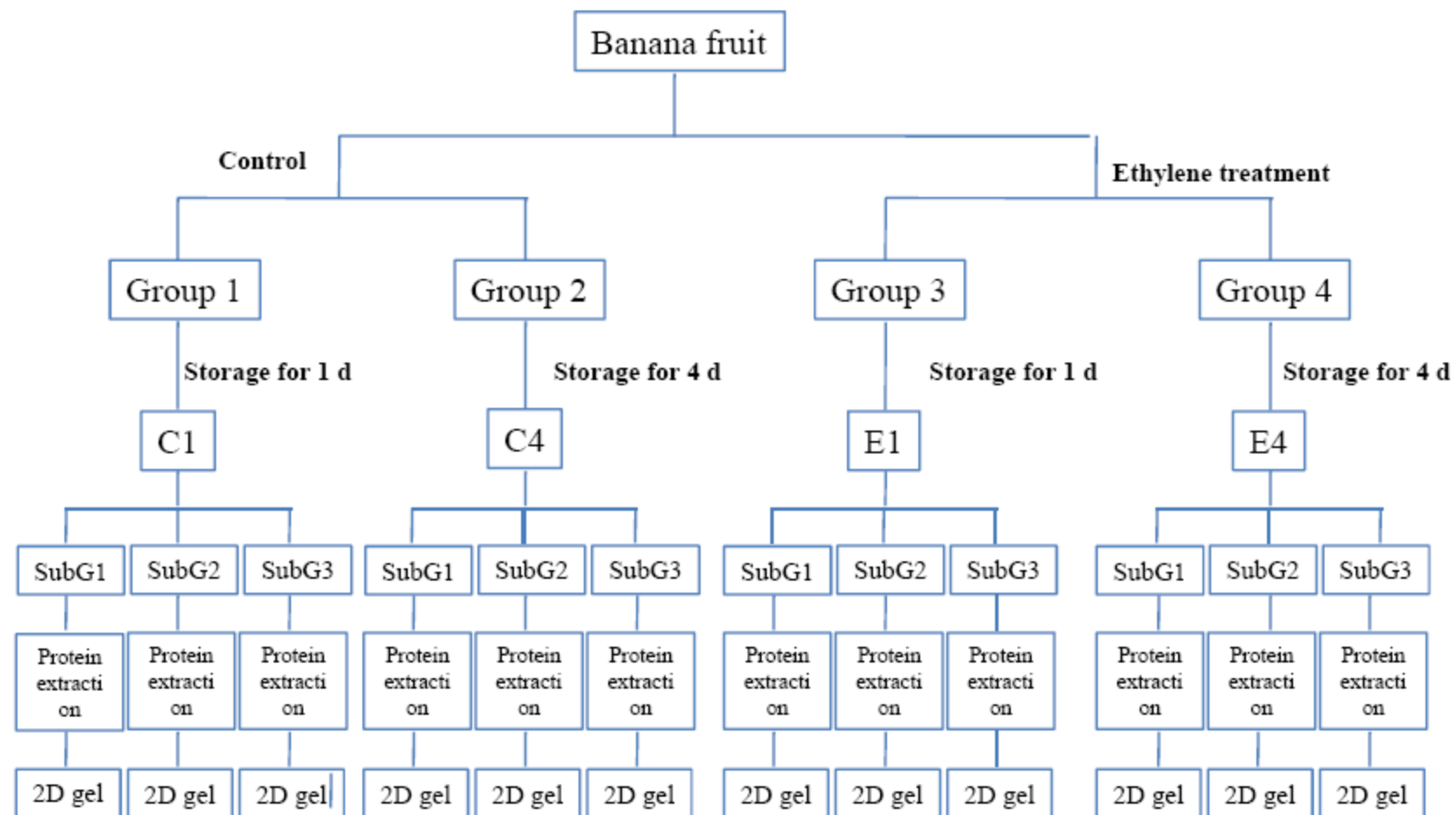

Figure S1

Supplement: Supplementary file 3 [file Image1.PDF]

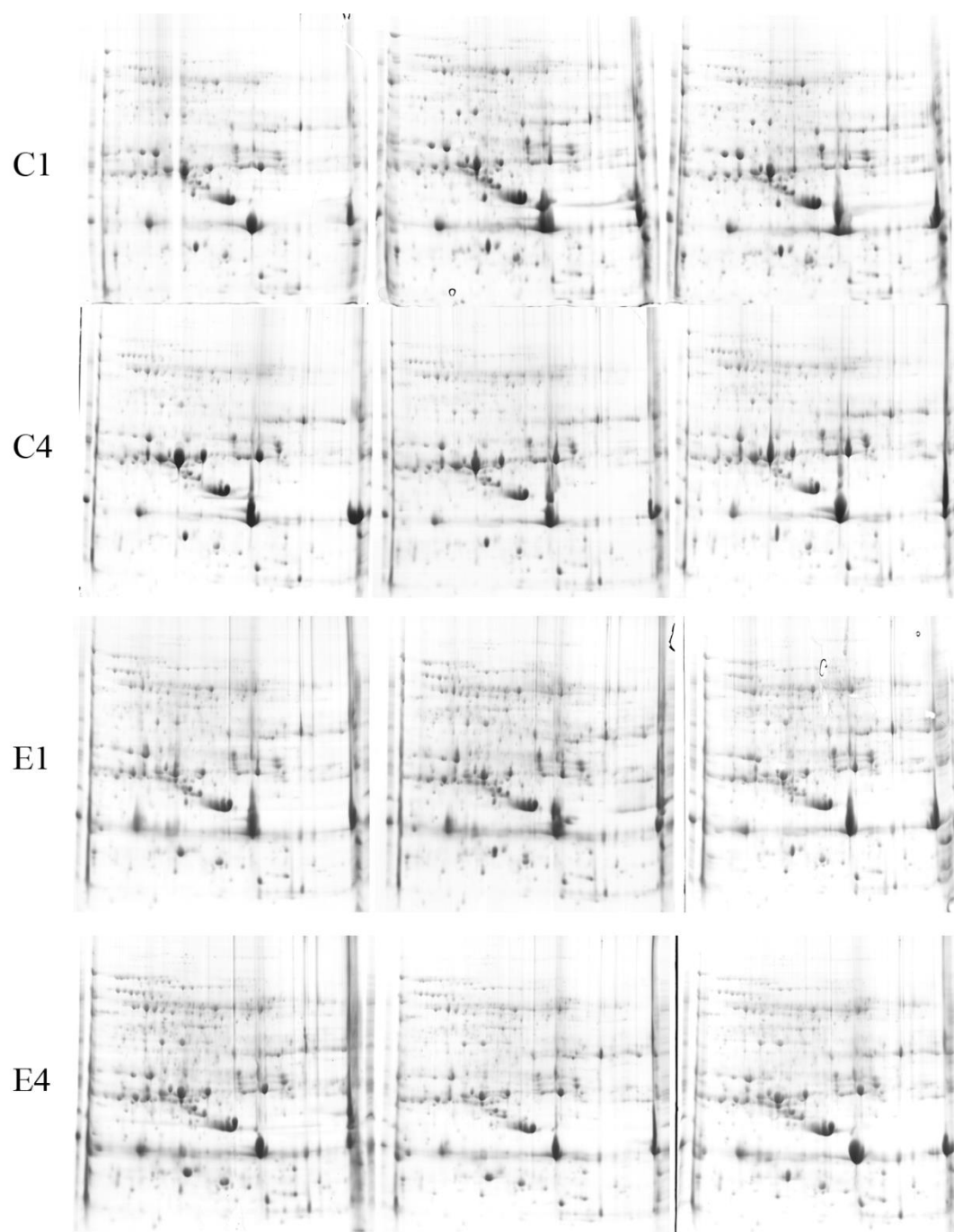

Figure S2

Supplement: Supplementary file 4 [file Image2.PDF]
